# Improvement of Flavonoids in Lemon Seeds on Oxidative Damage of Human Embryonic Kidney 293T Cells Induced by H_2_O_2_

**DOI:** 10.1155/2020/3483519

**Published:** 2020-04-19

**Authors:** Dingyi Yang, Yong Jiang, Yuqing Wang, Qianqian Lei, Xin Zhao, Ruokun Yi, Xin Zhang

**Affiliations:** ^1^Key Laboratory for Biorheological Science and Technology of Ministry of Education (Chongqing University), Chongqing University Cancer Hospital & Chongqing Cancer Institute & Chongqing Cancer Hospital, Chongqing 400044, China; ^2^Chongqing Collaborative Innovation Center for Functional Food, Chongqing Engineering Research Center of Functional Food, Chongqing Engineering Laboratory for Research and Development of Functional Food, Chongqing University of Education, Chongqing 400067, China; ^3^Chongqing Key Laboratory of Translational Research for Cancer Metastasis and Individualized Treatment, Chongqing University Cancer Hospital & Chongqing Cancer Institute & Chongqing Cancer Hospital, Chongqing 400030, China

## Abstract

In this study, flavonoids in lemon seeds (FLS) were used to assess its improvement on the oxidative damage of human embryonic kidney 293T cells (HEK 293T cells) induced by H_2_O_2_. *In vitro* experiments showed that the survival rates of HEK 293T cells treated with different flavonoid concentrations (50 *μ*g/mL, 100 *μ*g/mL, and 150 *μ*g/mL) exceeded 95%, indicating no significant toxic effect. Compared with the normal group, H_2_O_2_ (0.3 mmol/L) resulted significantly in oxidative stress injury of HEK 293T cells. The survival rate of the damaged cells increased after treatment with flavonoids, and the survival rate of cells treated with a high concentration (150 *μ*g/mL) of flavonoids was 76.2%. Flavonoids also effectively inhibited H_2_O_2_-induced apoptosis. At the same time, flavonoid treatment significantly reduced the malondialdehyde content in cells and increased the levels of catalase (CAT), superoxide dismutase (SOD), glutathione (GSH), and glutathione peroxidase (GSH-Px). Quantitative polymerase chain reaction (qPCR) and Western blot analysis also suggested that FLS upregulated mRNA and protein expressions of CAT, SOD (SOD1, SOD2), GSH (GSH1), and GSH-Px in H_2_O_2_-induced oxidative damage of HEK 293T cells. The high-performance liquid chromatography analysis demonstrated that FLS contained six compounds, including gallocatechin, caffeic acid, epicatechin, vitexin, quercetin, and hesperidin. FLS were proven to have a good antioxidant capacity *in vitro* and improve significantly the oxidative damage of HEK 293T cells induced by H_2_O_2_. The biological activity value warrants investigation in additional studies.

## 1. Introduction

*Citrus limon* (L.) Burm. f. has been widely consumed as a vitamin-rich fruit [1]. Most of the lemon seeds in the fruit are discarded. In traditional Chinese medicine, only a small amount of lemon seeds is added in some compounds in traditional Chinese medicines. Little is known regarding its biological activity in modern molecular biology and other technologies. One study has shown that lemon seed extract can play an antioxidant role in soybean oil processing [2]. Lemon seed extract can maintain oxidation stability and *α*-tocopherol in soybean oil processing [3]. *In vitro* experiments have demonstrated that lemon seed extract can inhibit the proliferation of human breast cancer and play an anticancer role [4].

Reactive oxygen species (ROS) produced by human aerobic metabolism mainly include superoxide anion (O_2_^−^), peroxy anion (O_2_^2-^), hydroxyl radical (·OH), organic peroxy radical (ROO^−^), and H_2_O_2_. When the amount of oxygen free radical (OFR) exceeds the range of its own antioxidant protection system and excessive oxidation occurs, it will attack human cells to obtain electrons and maintain its own stability, leading to damage relative to the conformation and the function of cells, as well as inflammation and other types of chronic diseases, such as cardiovascular diseases, nervous system diseases, kidney diseases, and cancer [5, 6]. The mechanisms of these diseases, for the most part, involve oxidative changes of key physiological molecules, such as regulation of proteins, lipids, carbohydrates, nucleic acids, gene expression, and inflammatory response [7]. To protect the body from damage of OFR, humans resist oxidative stress through their own antioxidant system and intake of exogenous antioxidants. The enzyme system includes superoxide dismutase (SOD), catalase (CAT), and the glutathione peroxidase (GSH-Px) system. Nonenzymatic antioxidants include gluten, tea polyphenols, tocopherols, flavonoids, and fatty acids [8]. As the main form of ROS *in vivo*, H_2_O_2_, with a wide range of sources and stable properties, can produce ·OH with intracellular Fe^2+^ via the Fenton reaction, thus causing a chain reaction, which is a preferred inducer for cell oxidative stress modeling [9].

293T cell is a human renal epithelial cell line, which exhibits an obvious influence on the external environment and strong protein expression. It is often used to examine the inhibition effect of oxidative stress of the test sample [10]. Renal epithelial cell damage is closely related to chronic renal diseases, such as chronic pyelonephritis and chronic obstructive nephropathy [11]. The damage of renal epithelial cells will lead to the decline of renal function and will also affect other diseases, including myeloma, leukemia, and malignant tumor [12]. Human embryonic kidney 293T can effectively detect the role of active substances in disease control through oxidative stress. Therefore, this study investigated the antioxidant activity of flavonoids *in vitro* and established an HEK 293T cell injury model using H_2_O_2_ as an inducer. Besides, the protective effect of different concentrations of flavonoids on cells with oxidative stress injury was evaluated. At the same time, the active components of flavonoids were determined, which provided reference for flavonoids to treat related diseases caused by oxidative stress.

## 2. Materials and Methods

### 2.1. Preparation for Extracts of Flavonoids from Lemon Seeds (FLS)

The extracts of FLS (Chongqing Huida Lemon Technology Group Co., Ltd, Chongqing, China) were dried to maintain a constant weight of lemon seeds, were ground in a mortar, and were sieved to obtain lemon seed powder. The powder was degreased with n-hexane for 90 min (degreasing temperature, 50°C; material liquid ratio, 1 : 30; and power, 200 W). After filtration and drying, flavonoids were extracted from 8 g of degreased lemon seed powder with 300 mL of 95% ethanol for 60 min (temperature, 50°C; power, 200 W). Finally, the sample solution was obtained by filtration and the dry FLS were evaporated using a rotary evaporator (Great Wall R-1050, Zhengzhou Greatwall Scientific Industrial and Trade Co., Ltd., Zhengzhou, Henan, China).

### 2.2. Cell Culture

Human embryonic kidney (HEK) 293T cells (Shanghai Institute of Biochemistry and Cell Biology, Shanghai, China) were resuscitated from liquid nitrogen and seeded in Dulbecco's Modified Eagle's Medium (DMEM) (high sugar, containing 10% fetal bovine serum and 1% penicillin-streptomycin double antibody solution) (Solarbio Life Sciences, Beijing, China). The medium was changed two or three times a week in a saturated humid environment at 37°C and 5% CO_2_. When the cell fusion reached 90%, trypsin (0.25%) (Solarbio Life Sciences) was used for digestion and passage. The cells in the logarithmic phase were used in all of the experiments.

### 2.3. Toxicity of FLS on HEK 293T Cells

HEK 293T cell suspension (1 × 10^4^ cells/mL) was seeded into a 96-well cell culture plate (60 *μ*L cells + 100 *μ*L culture medium), cultured at 37°C for 24 h until it adhered to the wall, and 20 *μ*L of FLS solution with different concentrations (normal group, 50 *μ*g/mL, 100 *μ*g/mL, and 150 *μ*g/mL) was added to the culture.

### 2.4. Detection of Cell Survival Rate by MTT

The MTT method was used to determine cell survival rate: HEK 293T cells were initially cultured on the wall for 24 h, with 20 *μ*L of FLS solution and with different concentrations (normal group, 50 *μ*g/mL, 100 *μ*g/mL, and 150 *μ*g/mL) for 24 h; 20 *μ*L of MTT (5 mg/mL) (Solarbio Life Sciences) was added, mixed, and cultured for 4 h. The upper medium was discarded, followed by adding 150 *μ*L of dimethyl sulfoxide (DMSO) (Solarbio Life Sciences), shaking for 30 min in dark period and at 37°C. Optical density (OD) was measured at 490 nm. The experiment was performed in triplicate. Cell survival rate (%) = (Ar/As) × 100%, where Ar is OD of the FLS treatment group and As is OD of the normal group (Evolution™ 350, Thermo Fisher Scientific, New York, USA).

### 2.5. Observation of Cell Proliferation

**Figure 1:**
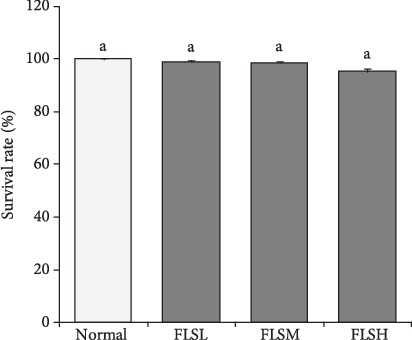
Effect of treatment with flavonoids of lemon seeds (FLS) on the survival rate of human embryonic kidney 293T cells. Values presented are the mean ± standard deviation (*N* = 6/group). ^a-e^Mean values with different letters over the bar are significantly different (*P* < 0.05) according to Tukey's honestly significant difference. Normal: untreated HEK 293T cells; FLSL: 50 *μ*g/mL of FLS-treated HEK 293T cells; FLSM: 100 *μ*g/mL of FLS-treated HEK 293T cells; FLSH: 150 *μ*g/mL of FLS-treated HEK 293T cells.

HEK 293T cell suspension (1 × 10^4^ cells/mL) was seeded into a 96-well cell culture plate (60 *μ*L cells + 100 *μ*L culture medium), cultured at 37°C for 24 h, adhered to the wall, and cultured with 20 *μ*L of H_2_O_2_ (0.3 mmol/L), and 20 *μ*L of FLS solution with different concentrations (normal group, 40 *μ*g/mL, 100 *μ*g/mL, and 160 *μ*g/mL) was added to the culture for 24 h, and the cell survival rate was measured using the MTT method (Figure 1(a)).

HEK 293T cell suspension (1 × 10^4^ cells/mL) was seeded into a 96-well cell culture plate (60 *μ*L cells + 100 *μ*L culture medium), cultured at 37°C for 24 h, adhered to the wall, and cultured with 20 *μ*L of H_2_O_2_ (0.3 mmol/L) for 4 h to prepare the oxidative damage model. After discarding the upper medium, 20 *μ*L of FLS solution with different concentrations (normal group, 40 *μ*g/mL, 100 *μ*g/mL, and 160 *μ*g/mL) was added to the culture for 24 h, and the cell survival rate was measured using the MTT method (Figure 1(b)).

### 2.6. Observation of Apoptosis by Flow Cytometry

HEK 293T cells were treated according to the cell proliferation experiment, collected, and formed into single cell suspension. After fixing and staining, apoptosis was detected using flow cytometry (Accuri C6, BD Biosciences, San Jose, CA, USA).

### 2.7. Determinations of Malondialdehyde (MDA), SOD, GSH, GSH-Px, and CAT in HEK 293T Cells

HEK 293T cells in logarithmic growth phase were digested with 0.25% trypsin, seeded in a six-well cell culture plate (1 × 10^5^ cells/mL), cultured with 2 mL of DMEM in a saturated humid environment at 37°C and 5% CO_2_ for 24 h, adhered to the wall, with the addition of 200 *μ*L of H_2_O_2_ (0.3 mmol/L), mixed well, and cultured for 4 h to prepare the oxidative damage model. FLS aqueous extract (200 *μ*L) with different concentrations (0 *μ*g/mL, 50 *μ*g/mL, 100 *μ*g/mL, and 150 *μ*g/mL) was added into each well of the HEK 293T cell model of oxidative damage, and PBS solution (0.1 mol/L) was added to maintain balance. The cells were further cultured at 37°C and 5% CO_2_ for 24 h. HEK 293T cells treated with FLS were removed from the old culture medium, washed with precooled PBS, detached from the wall with 200 *μ*L of trypsin, transferred to a 1.5 mL centrifuge tube, and centrifuged to discard the supernatant. The cells were again washed with precooled PBS and centrifuged at 4,000 r/min for 15 min to discard the supernatant, with the addition of 800 *μ*L normal saline, and homogenized. The content of MDA, SOD, GSH, GSH-Px, and CAT in the homogenate was determined according to the kit instructions (Nanjing Jiancheng Bioengineering Institute, Nanjing City, China).

### 2.8. Clonogenic Assay

The HEK 293T cells were cultured according to the cell culture method in Section 2.2; the HEK 293T cells in the logarithmic growth stage were digested by 2.5 g/L trypsin to form a single cell suspension. The HEK 293T cells were inoculated into 24-well plates, with 50 cells per well. Each group of cells was inoculated with six wells, five groups in total (normal, control, FLSL, FSLM, and FLSH groups). HEK 293T cells in the normal group were cultured for 1 week without any other treatment. HEK 293T cells in the control group were cultured using 900 *μ*L DMEM culture medium and 100 *μ*L culture medium solution containing H_2_O_2_ (0.3 mmol/L); the FLSL group, FSLM group, and FLSH group were cultured using 800 *μ*L DMEM culture medium, 100 *μ*L culture medium solution containing H_2_O_2_ (0.3 mmol/L), and 100 *μ*L culture medium solution containing FLS (40 *μ*g/mL, 80 *μ*g/mL, and 160 *μ*g/mL) for 1 week. The culture medium was discarded, the wells of the culture dish were washed using PBS, fixed with 1 mL pure methanol for 15 min, and Giemsa stain solution (Solarbio Life Sciences) was used for dyeing. The number of clones comprising more than 50 cells was counted when the culture plate was placed in the low multiple of microscope (IX73, Olympus, Tokyo, Japan), and the clone formation rate was calculated according to the following formula: clone formation rate (%) = clone number/inoculation number × 100% [13].

### 2.9. Quantitative Polymerase Chain Reaction (qPCR)

HEK 293T cells were cultured into a six-well plate (1 × 10^5^ cells/mL) and treated with FLS at different concentrations after modeling. The total RNA of HEK 293T cells was extracted using the TRIzol Reagent (Thermo Fisher Scientific). We added 1 *μ*L of Oligo (dT) 18 primer (500 ng) and 1.0 *μ*L of total RNA (1.0 *μ*g) to 10.0 *μ*L of nonnuclease water heated at 65°C for 5 min on a gradient PCR apparatus. Then, the mixture containing 4.0 *μ*L of 5x reaction buffer, 1.0 *μ*L of RiboLock RNase Inhibitor (20 U), 2.0 *μ*L of 10 mM dNTP Mix, and 1.0 *μ*L of RevertAid Reverse Transcriptase (200 U/*μ*L) (Thermo Fisher Scientific) was added to the total RNA system, which was transcribed into cDNA at 42°C for 60 min and 70°C for 5 min. Amplification conditions were as follows: denaturation at 95°C for 3 min, annealing at 60°C for 30 s, extension at 95°C for 1 min, and a total of 40 cycles. mRNA expressions of SOD, CAT, GSH, and GSH-Px were detected using qPCR (Table 1). cDNA of each gene was amplified three times in parallel, and the mean of Ct was obtained. The housekeeping gene GAPDH was used as an internal reference, and related genes were calculated according to 2^−*∆∆*Ct^ (StepOnePlus, Thermo Fisher Scientific) [14].

### 2.10. Western Blot

After cell lysis using RIPA cell lysis buffer, the supernatant was isolated, in which the protein concentration was measured using a protein detection kit. We isolated 30–50 *μ*g protein by SDS-PAGE, and we electroimprinted it onto a nitrocellulose (NC) membrane. The NC membrane was sealed and, in turn, bound with primary antibodies and secondary antibodies of SOD1, SOD2, GSH1, and CAT, and the antibody (Thermo Fisher Scientific) binding zone was detected using chemiluminescence (Tanon Science and Technology Co., Ltd., Shanghai, China) [15].

### 2.11. High-Performance Liquid Chromatography (HPLC)

The FLS extracts were dissolved in DMSO to obtain a solution with a concentration of 10 mg/mL, and then diluted with 50% methanol to obtain a final concentration of 2 mg/mL. After passing through a 0.22 *μ*m organic filter membrane, the solution was tested with an injection volume of 5 *μ*L. The data were as follows: chromatographic column: Accucore C18 column (2.6 *μ*m, 4.6 mm × 150 mm); mobile phase A: 0.5% acetic acid; mobile phase B: acetonitrile; flow rate: 0.6 mL/min; column temperature: 35°C; detection wavelength: 285 nm; gradient elution conditions: 0–10 min, 12%–25% A; 10–30 min, 25%–45% B.

### 2.12. Statistics

SPSS 20.0 statistical software was used to analyze the data. All of the experimental data were repeated three times. The results are expressed as mean ± standard deviation values. The results were analyzed using Duncan's multirange test and one-way analysis of variance. A *P* < 0.05 was considered to be statistically significant.

## 3. Results

### 3.1. Toxicity of FLS on HEK 293T Cells

As shown in Figure 1, after treatment by 50 *μ*g/mL, 100 *μ*g/mL, and 150 *μ*g/mL of FLS extracts, the survival rates of HEK 293T cells exceeded 95%, indicating no significant lethal effect of FLS within this range (0–150 *μ*g/mL) on HEK 293T cells. Therefore, FLS extracts from lemon seed with concentrations of 50 *μ*g/mL, 100 *μ*g/mL, and 150 *μ*g/mL were used in the subsequent studies.

### 3.2. Protective Effect of FLS on Proliferation of H_2_O_2_-Induced HEK 293T Cells

**Figure 2:**
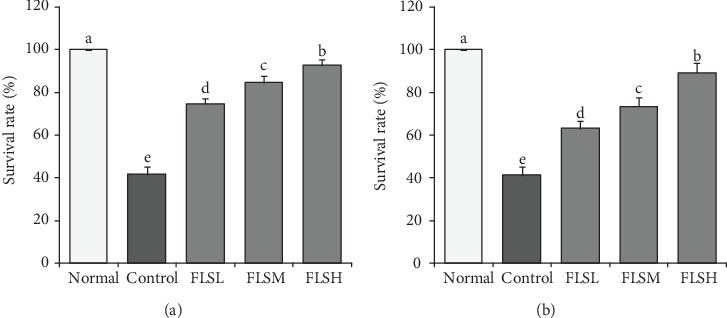
Effect of flavonoids of lemon seeds (FLS) on the survival rate of H_2_O_2_-damaged human embryonic kidney 293T cells. (a) Simultaneous treatment with H_2_O_2_ and FLS; (b) treatment with FLS after H_2_O_2_-induced oxidative damage. ^a-e^Mean values with different letters over the bar are significantly different (*P* < 0.05) according to Tukey's honestly significant difference. Normal: untreated HEK 293T cells; FLSL: 50 *μ*g/mL of FLS-treated HEK 293T cells; FLSM: 100 *μ*g/mL of FLS-treated HEK 293T cells; FLSH: 150 *μ*g/mL of FLS-treated HEK 293T cells.

As shown in Figure 2, compared with normal cells, the survival rate of HEK 293T cells injured by H_2_O_2_ decreased significantly. After simultaneous treatment with H_2_O_2_ (Figure 2(a)) and after H_2_O_2_-induced oxidative damage (Figure 2(b)), the survival rates of the cells treated with 50 *μ*g/mL, 100 *μ*g/mL, and 150 *μ*g/mL FLS extracts were significantly improved, and the survival rate of the cells treated with high concentration (150 *μ*g/mL) was 89.2%, with more significant effect (*P* < 0.05).

### 3.3. Inhibition of FLS on Damage of HEK 293T Cells Induced by H_2_O_2_

**Figure 3:**
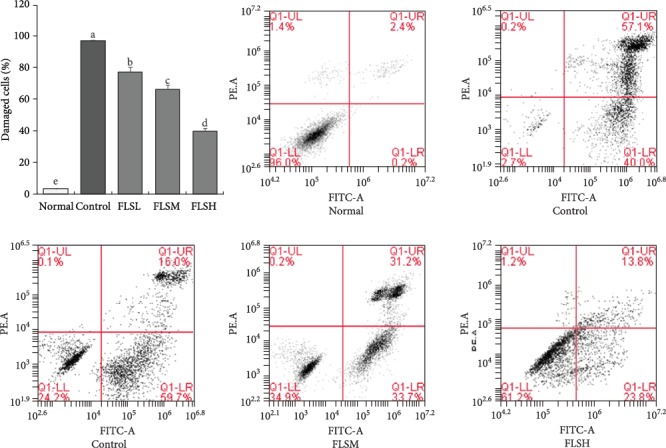
Inhibitory apoptosis effect of flavonoids of lemon seeds (FLS) on H_2_O_2_-damaged human embryonic kidney 293T cells. ^a-e^Mean values with different letters over the bar are significantly different (*P* < 0.05) according to Tukey's honestly significant difference. Normal: untreated HEK 293T cells; FLSL: 50 *μ*g/mL of FLS-treated HEK 293T cells; FLSM: 100 *μ*g/mL of FLS-treated HEK 293T cells; FLSH: 150 *μ*g/mL of FLS-treated HEK 293T cells.

In Figure 3, compared with normal cells, severe damage (damaged and apoptosis cells) is shown in HEK 293T cells injured by H_2_O_2_ (97.1% damaged and apoptosis cells in the control group). After treatment with 50 *μ*g/mL (77.1% damaged and apoptosis cells), 100 *μ*g/mL (66.2% damaged and apoptosis cells), and 150 *μ*g/mL FLS (39.8% damaged and apoptosis cells) extracts, cell damage is significantly inhibited (*P* < 0.05), and the inhibitory effect is better with increased FLS concentration.

### 3.4. Changes in MDA, SOD, GSH, GSH-Px, and CAT Levels in HEK 293T Cells Treated with FLS

**Figure 4:**
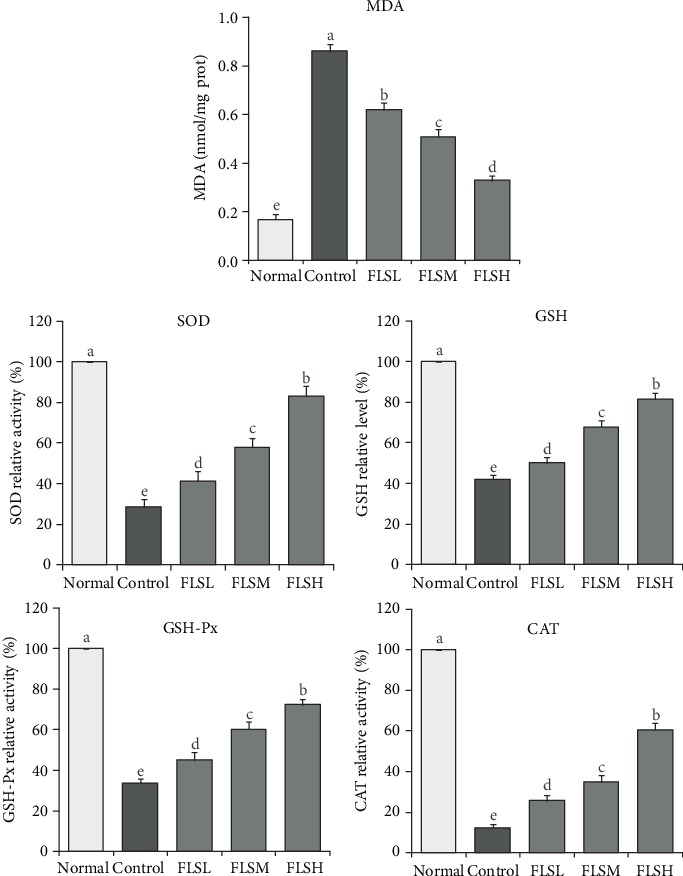
Effect of flavonoids of lemon seeds (FLS) on the levels of MDA, SOD, GSH, GSH-Px, and CAT in human embryonic kidney 293T cells exposed to H_2_O_2_. ^a-e^Mean values with different letters over the bar are significantly different (*P* < 0.05) according to Tukey's honestly significant difference. Normal: untreated HEK 293T cells; FLSL: 50 *μ*g/mL of FLS-treated HEK 293T cells; FLSM: 100 *μ*g/mL of FLS-treated HEK 293T cells; FLSH: 150 *μ*g/mL of FLS-treated HEK 293T cells.

As shown in Figure 4, the MDA content of HEK 293T cells treated by H_2_O_2_ (0.3 mmol/L) for 4 h is significantly higher than that in normal cells, but the OD, GSH, GSH-Px, and CAT levels are significantly lower. After being treated with FLS at different concentrations (50 *μ*g/mL, 100 *μ*g/mL, and 150 *μ*g/mL), the MDA content is significantly lower (*P* < 0.05), but the SOD, GSH, GSH-Px, and CAT levels are significantly higher (*P* < 0.05). The effect of 150 *μ*g/mL FLS treatment is the most significant.

### 3.5. Effects of FLS on Clone Formation in Damaged HEK 293T Cells

As shown in Table 2, compared to the normal HEK 293T cells, H_2_O_2_ had a significant effect (*P* < 0.05) on cell clone formation of HEK 293T cells, and the control group had the least number of HEK 293T cell clones. FLS could significantly (*P* < 0.05) inhibit the decrease of clonal formation caused by H_2_O_2_, and with the increase of FLS concentration, the inhibition effect was stronger.

### 3.6. Effects of FLS on Expressions of SOD, CAT, GSH, and GSH-Px in Damaged HEK 293T Cells

**Figure 5:**
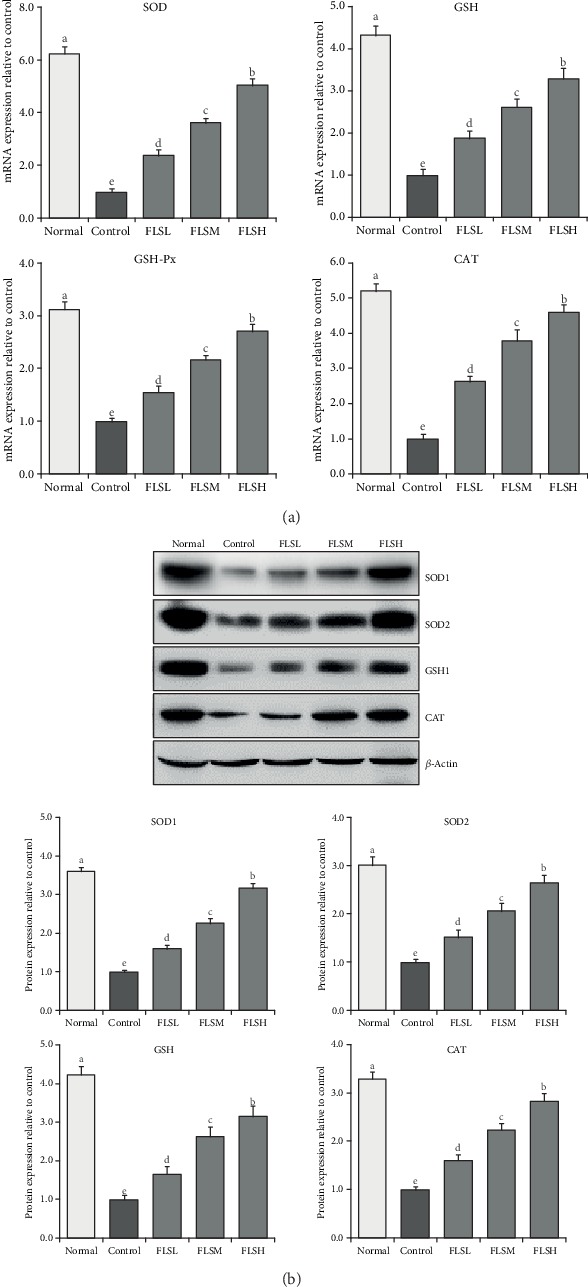
mRNA (a) and protein (b) expression of flavonoids of lemon seeds (FLS) on H_2_O_2_-damaged human embryonic kidney 293T cells. ^a-e^Mean values with different letters over the bar are significantly different (*P* < 0.05) according to Tukey's honestly significant difference. Normal: untreated HEK 293T cells; FLSL: 50 *μ*g/mL of FLS-treated HEK 293T cells; FLSM: 100 *μ*g/mL of FLS-treated HEK 293T cells; FLSH: 150 *μ*g/mL of FLS-treated HEK 293T cells.

As shown in Figure 5, after H_2_O_2_-induced injury (0.3 mmol/L), the mRNA expressions of SOD, CAT, GSH, and GSH-Px in HEK 293T cells and the protein expressions of SOD1, SOD2, CAT, and GSH are significantly decreased. SOD, CAT, GSH, and GSH-Px expressions in the damaged cells are significantly increased after treatment with FLS (*P* < 0.05), which is consistent with the results of the kit test. FLS could increase the SOD, CAT, GSH, and GSH-Px levels as it was determined using a kit to determine the expression in damaged HEK 293T cells using qPCR and Western blot assays.

### 3.7. Determination of FLS by HPLC

**Figure 6:**
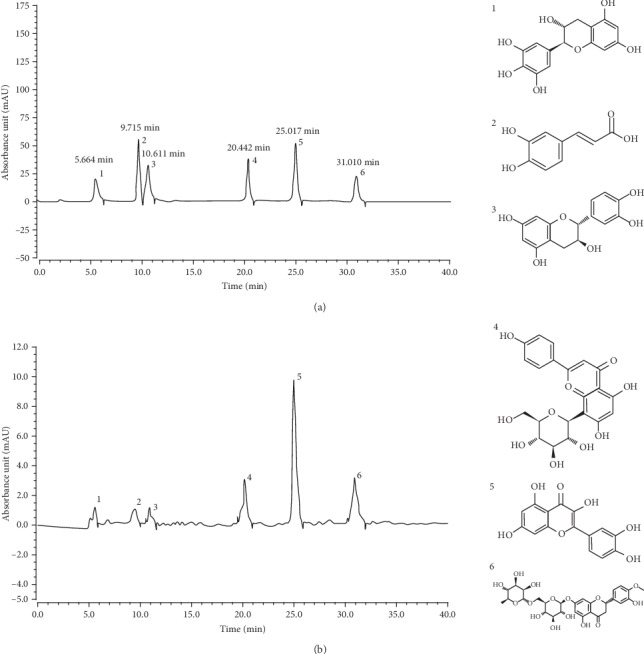
Flavonoid extract constituents of lemon seeds: (a) standard chromatograms; (b) flavonoids of lemon seed chromatograms. 1: gallocatechin; 2: caffeic acid; 3: epicatechin; 4: vitexin; 5: quercetin; 6: hesperidin.

As shown in Figure 6, FLS contain six compounds, including gallocatechin, caffeic acid, epicatechin, vitexin, quercetin, and hesperidin. Their peak retention time on the liquid chromatogram was 5.664 min, 9.715 min, 10.611 min, 20.442 min, 25.017 min, and 31.010 min, respectively. The contents of the six components in the FLS were 18.304 mg/g, 37.294 mg/g, 25.080 mg/g, 108.539 mg/g, 334.585 mg/g, and 145.983 mg/g, respectively.

## 4. Discussion

The damage caused by oxidative stress affected the proliferation of normal cells and led to cell death and decline in the proliferation of cells [16]. Thus, FLS can inhibit the decreased proliferation of HEK 293T cells induced by H_2_O_2_ and protect normal cell proliferation. Oxidative stress can damage cells, cause apoptosis, promote death of normal cells, and effectively protect normal cells by reducing the degree of apoptosis of oxidation-damaged cells [17]. When FLS and H_2_O_2_ are added to the cultured cells at the same time, FLS can react directly with H_2_O_2_, reducing the amount of H_2_O_2_ that can damage the cells, thus playing a stronger role than the intervention of adding the FLS after H_2_O_2_ first damages the cells. Therefore, FLS can effectively prevent cell apoptosis caused by oxidative stress and protect normal cells.

MDA released from cell membrane reacts with protein and nucleic acid, causes crosslinking polymerization, and inhibits protein synthesis, mainly by damaging membrane structure and function, changing its permeability, thus affecting biochemical reaction in the normal body [18]. Therefore, MDA content can reflect the degree of lipid peroxidation and cell damage. The intake of appropriate antioxidant substances can reduce the degree of ROS-induced lipid peroxidation [19]. CAT, SOD, and GSH-Px are important antioxidant enzymes, and GSH is also the one with good antioxidant effect. They can effectively regulate oxidative balance in the body and inhibit the damage caused by oxidative stress [20]. The results of this study indicate that FLS effectively reduce the oxidative stress damage caused by H_2_O_2_ on cells and protect cells.

Clonogenic analysis (colony formation analysis) is an *in vitro* cell survival test based on the ability of a single cell to grow into a colony, which is essentially an experiment of the “infinite” dividing power of each cell in a population. Clonogenic assays can be used to determine the cytotoxicity or the efficacy of drugs or active substances [21]. In this study, we also found that FLS could effectively inhibit the abnormal clone formation of normal cells caused by hydrogen peroxide to achieve the effect of cell protection.

Epigallocatechin, which is a compound derived from plants, has certain antioxidant activity [22]. Caffeic acid has a wide range of antibacterial and antiviral activities [23] and has been proven to inhibit the formation of lipid peroxides in the murine brain homogenate [24]. Epicatechin can function as antioxidants, scavenging free radicals, strengthening metabolism, and regulating immunity and antitumor activity. Its antioxidant capacity is considered to be the combination of phenolic hydroxyl groups and free radicals in molecules to scavenge free radicals [25]. The main function of vitexin is to promote blood circulation, remove blood stasis, regulate qi, and dredge blood vessels. Vitexin is also a natural drug component for cancer prevention and antitumor activity [26]. Quercetin, which is widely found in plants, has been shown to have antioxidant effect, anti-inflammatory effect, antiviral effect, antitumor activity, antiatherosclerosis effect, neuroprotective effect, blood pressure lowering efficacy, and other biological activities [27]. Hesperidin, which is anti-inflammatory and antiviral with antibacterial effects, is used for treating hypertension and myocardial infarction [28]. These six substances are all compounds with effective biological activity. The normal protective effect of FLS is attributed to the biological activity of these chemicals, and the mixture of these compounds may produce an improved combined effect, which warrants additional investigation.

## 5. Conclusions

In this study, *in vitro* experiments were conducted to evaluate the effect of FLS on the improvement in cell level and potential active compounds under oxidative damage. The results of *in vitro* experiments showed that FLS demonstrated beneficial effects on the oxidative cell damage, which avoided cell death caused by oxidative stress, inhibited cell apoptosis caused by oxidative damage, and enhanced the antioxidant enzymes in cells to resist oxidative stress. The component analysis results indicated that the active compounds contained in FLS had multiple biological activities, and that their combination showed an inhibitory effect on oxidative cell damage. However, this study only preliminarily investigated antioxidant activity and improvement on the oxidative damage of FLS from lemon seeds *in vitro*. The mechanism of antioxidant protection *in vivo* requires additional exploration.

## Figures and Tables

**Table 1:** Sequences of reverse transcription-polymerase chain reaction primers.

Accession number	Gene name	Sequence
NM_000454.4	*SOD*	Forward: 5′-AGATGGTGTGGCCGATGTGT-3′ Reverse: 5′-TCCAGCGTTTCCTGTCTTTGTA-3′

NM_001752.3	*CAT*	Forward: 5′-TGTTGCTGGAGAATCGGGTTC-3′ Reverse: 5′-TCCCAGTTACCATCTTCTGTGTA-3′

NM_000178.4	*GSH*	Forward: 5′-TACGGCTCACCCAATGCTC-3′ Reverse: 5′-CTATGGCACGCTGGTCAAATA-3′

NM_201397.2	*GSH-Px*	Forward: 5′-GTCGGTGTATGCCTTCTCGG-3′ Reverse: 5′-CTGCAGCTCGTTCATCTGGG-3′

NM_002046.7	*GAPDH*	Forward: 5′-TCAAGAAGGTGGTGAAGCAGG-3′ Reverse: 5′-AGCGTCAAAGGTGGAGTG-3′

**Table 2:** Effects of flavonoids of lemon seeds (FLS) on clone formation in H_2_O_2_ induced damage human embryonic kidney 293T cells.

Group	Number of clones	Clonogenic rate (%)
Normal	48.39 ± 4.57^a^	100.00 ± 0.00^a^
Control	10.32 ± 3.02^e^	21.33 ± 2.77^e^
FLSL	20.59 ± 3.80^d^	42.55 ± 3.12^d^
FLSM	31.14 ± 4.11^c^	64.35 ± 4.83^c^
FLSH	39.67 ± 2.03^b^	82.00 ± 2.46^b^

Values presented are the mean ± standard deviation (*N* = 6/group). ^a-e^Mean values with different letters over the same column are significantly different (*P* < 0.05) according to Tukey's honestly significant difference. Normal: untreated HEK 293T cells; FLSL: 50 *μ*g/mL of FLS-treated HEK 293T cells; FLSM: 100 *μ*g/mL of FLS-treated HEK 293T cells; FLSH: 150 *μ*g/mL of FLS-treated HEK 293T cells.

## Data Availability

No data were used to support this study.
